# FOLFIRINOX in advanced pancreatic cancer patients with the double-variant type of UGT1A1 *28 and *6 polymorphism: a multicenter, retrospective study

**DOI:** 10.1007/s00280-020-04206-w

**Published:** 2021-01-02

**Authors:** Kumiko Umemoto, Hideaki Takahashi, Chigusa Morizane, Ikuhiro Yamada, Satoshi Shimizu, Kazuhiko Shioji, Yukio Yoshida, Masayo Motoya, Nobumasa Mizuno, Yasushi Kojima, Takeshi Terashima, Kazuhiro Uesugi, Makoto Ueno, Junji Furuse, Tetsuo Akimoto, Masafumi Ikeda

**Affiliations:** 1 Department of Hepatobiliary and Pancreatic Oncology, National Cancer Center Hospital East, 6-5-1 Kashiwanoha, Kashiwa, Chiba 277-8577 Japan; 2 Course of Advanced Clinical Research of Cancer, Juntendo University Graduate School of Medicine, Bunkyo-ku, Tokyo, Japan; 3 Department of Gastroenterology, Cancer Institute Hospital, Japanese Foundation For Cancer Research, Tokyo, Japan; 4 Department of Gastroenterology, Saitama Cancer Center, Saitama, Japan; 5 Department of Internal Medicine, Niigata Cancer Center Hospital, Niigata, Japan; 6 Division of Gastrointestinal Oncology, Shizuoka Cancer Center, Shizuoka, Japan; 7 Department of Gastroenterology and Hepatology, Sapporo Medical University, Sapporo, Japan; 8 Department of Gastroenterology, Aichi Cancer Center Hospital, Aichi, Japan; 9 Department of Gastroenterology, National Center for Global Health and Medicine, Tokyo, Japan; 10 Department of Gastroenterology, Kanazawa University Hospital, Kanazawa, Japan; 11 Departments of Gastroenterology, National Hospital Organization Shikoku Cancer Center, Matsuyama, Japan; 12 Department of Gastroenterology, Hepatobiliary and Pancreatic Medical Oncology Division, Kanagawa Cancer Center, Yokohama, Japan; 13 Faculty of Medicine, Department of Medical Oncology, Kyorin University, Tokyo, Japan; 14 Department of Radiation and Oncology and Particle Therapy, National Cancer Center Hospital East, Kashiwa, Japan

**Keywords:** UGT1A1 *28 or *6 polymorphism, Advanced pancreatic cancer, Irinotecan

## Abstract

**Background:**

UGT1A1 *28 and *6 polymorphism is associated with reduced enzyme activity and severe toxicities of irinotecan, especially in patients with homozygous or heterozygous for UGT1A1*28 or *6 polymorphism for both UGT1A1*28 and *6 (double-variant-type of UGT1A1 polymorphism, UGT1A1-DV). FOLFIRINOX is one of the standard treatments for metastatic pancreatic cancer (PC). The optimal dose of irinotecan as a component of the FOLFIRINOX has not been established yet for patients with UGT1A1-DV.

**Patients and methods:**

Advanced PC patients with UGT1A1-DV who had received at least one cycle of FOLFIRINOX from December 2013 to March 2016 were collected retrospectively conducted at multicenter in Japan. We evaluated the patient characteristics, efficacy and safety of FOLFIRINOX and investigate the optimal initial dose of irinotecan in Japanese advanced PC patients with UGT1A1-DV.

**Results:**

A total of 31 patients were enrolled. Grade 4 neutropenia was seen more frequently (67%; 4/6) in patients who had received irinotecan at an initial dose of  ≥ 150 mg/m^2^ than in those who had received the drug at an initial dose of  ≤ 120 mg/m^2^ (20%; 5/24). The response rate (RR) and progression-free survival (PFS) in patients given irinotecan of  ≤ 120 mg/m^2^ were 21.4% and 8.1 months, respectively, which were consistent with previous report for patients without UGT1A1-DV.

**Conclusion:**

Based on our findings, we recommend that in Japanese advanced PC patients with UGT1A1- DV treated with FOLFIRINOX, irinotecan be administered at an initial dose of  ≤ 120 mg/m^2^.

## Introduction

Pancreatic cancer (PC) carries poor prognosis and the reported 5-year survival rates of patients with PC are in the dismal range of 4–7% [1, 2]. PC is the fourth leading cause of death from cancer in Japan, with the mortality number increasing every year [3]. Some of the established standard chemotherapies in Japan for stage IV (UICC 7^th^ edition), referred to as metastatic PC include gemcitabine (GEM) [4], S-1 [5], GEM + erlotinib [6], GEM + nab-paclitaxcel (nab-PTX) [7], and FOLFIRINOX in Japan. In the PRODIGE 4 trial [8], Conroy et al. demonstrated that superior efficacy and safety of FOLFIRINOX to those of GEM alone as first-line therapy among patients with metastatic PC. FOLFIRINOX, which consists of oxaliplatin, leucovorin, irinotecan, and 5-fluorouracil (5-FU), administered by intravenous bolus, followed by a continuous intravenous infusion of 5-FU over a 46-h period every 2 weeks considered as standard dose of therapy. FOLFIRINOX was demonstrated to yield an impressive response rate (RR) of 31.6%, with a significantly improved overall survival (OS) (median, 11.1 months) as compared to GEM alone. Ever since, FOLFIRINOX has become one of the standard treatments for metastatic PC, with a good PS, in North America and Europe. Okusaka et al. reported a phase II study of full dose of FOLFIRINOX [9] in Japanese patients with advanced PC, which showed similar efficacy to that in the previous phase III trial, with a high incidence of in grade 3 or more severe neutropenia (77.8%) and febrile neutropenia (FN) (22.2%) in chemotherapy-naïve Japanese patients with metastatic PC. Ozaka et al. reported a phase II trial of modified FOLFIRINOX (mFOLFIRINOX) attempted to improve the tolerability as a Japanese phase II trial [10], in which they eliminated the intravenous bolus injection of 5-FU and reduced the irinotecan dose to 150 mg/m^2^. In this study, the incidence of grade 3 or severe neutropenia was 47.8%, and that of FN was 8.7%, which were less than those in the previously reported Japanese full-dose phase II study, without any negative impact on the efficacy; the OS and RR were 11.2 months and 37.7%, respectively. Therefore, mFOLFIRINOX is commonly used in Japan for patients with advanced PC.

Irinotecan, which is the one of major component drugs consisting of FOLFIRINOX therapy, is a semisynthetic camptothecin derivative with topoisomerase 1-inhibiting activity, and is a prodrug, activated by a carboxylesterases to SN-38 [11]. It is also converted to an its inactive metabolite, SN-38 glucuronide (SN-38G), by uridine disphosphate-glucuronosyl-transferase (UGT) in the liver and eliminated in the feces. There are some variabilities in the expression of UGT and UGT1A1 is the main genetic polymorphisms of UGT. Patients with homozygous for UGT1A1*28 or *6 and heterozygous for both UGT1A1*28 and *6 (double-variant-type UGT1A1 polymorphism, UGT1A1-DV) show a decreased ability to metabolize irinotecan, and consequently, an increased risk of severe toxicities [12]. UGT1A1-DV includes the following three UGT1A1 genotypes: *28/*28, *6/*6, and *28/*6. The reported frequency of the UGT1A1 *28 is 30–40% in western countries and 15% in Asian countries, and, that of UGT1A1 *6 is 0–1% in western countries and 8.5–11% in Asian countries, thus the frequencies of both differed between the races.

Prior two studies did not discuss the subset of patients with UGT1A1-DV; patients with UGT1A1-DV were not prescribed in the PRODIGE 4 study [8], and the study of metastatic PC patients conducted in Japan excluded patients with UGT1A1-DV [9]. Therefore, the initial dose of irinotecan as a component of the FOLFIRINOX regimen has not yet been established for patients with UGT1A1-DV. We hypothesized that patients with the UGT1A1-DV genotypes may need to be prescribed reduced doses of irinotecan, as compared to the standard dose, to ensure safety. The purpose of this study was to determine the optimal initiating dose of irinotecan and evaluate the safety and efficacy of FOLFIRINOX in Japanese advanced PC with UGT1A1-DV.

## Patients and methods

### Patients

The criteria for inclusion in this study were (1) histologically proven diagnosis of ductal pancreatic adenocarcinoma or adenosquamous carcinoma, (2) unresectable or recurrent disease, (3) treatment any line with FOLFIRINOX between December 2013 and March 2016, (4) expression of UGT1A1-DV, and (5) administration of the first cycle of FOLFIRINOX had been completed. UGT1A1*6 and *28 genotype were checked in all patients with pancreatic cancer at the 16 centers before the initiation of FOLFIRINOX. Every patient with UGT1A1-DV was consecutively registered in this study.

### Treatment

The full dose of FOLFIRINOX regimen consisted of oxaliplatin, irinotecan, leucovorin, and 5-FU bolus plus 46-h infusion biweekly [8] (oxaliplatin 85 mg/m^2^, leucovorin 200 mg/m^2^, irinotecan 180 mg/m^2^, and 5-FU bolus 400 mg/m^2^, followed by continuous intravenous infusion of 2400 mg/m^2^ over a 46-h period every 2 weeks). The initial doses were modified at the discretion of the treating physician.

### Assessment

This was a retrospective, multicenter study conducted in Japan and the clinical data of the patients were obtained from their electronic medical records. The collected clinical data included the Eastern Cooperative Oncology Group performance status (ECOG PS [13]), primary and metastatic sites at diagnosis, history of previous surgery and adjuvant chemotherapy, start and stop date of FOLFIRINOX, type and severity of adverse events and dose reductions, response to first-line therapy, date of progression, and date of death.

Adverse events during the whole cycles of FOLFIRINOX were recorded according to the National Cancer Institute’s common terminology criteria for adverse events v4.0 (CTCAE 4.0); the incidence of severe adverse events was also evaluated during the first two cycles of FOLFIRINOX. Severe adverse events were also defined as (1) grade 4 of neutropenia sustaining for more than 8 days, (2) febrile neutropenia (FN) or grade 3 or worse of neutropenia with infection, (3) grade 4 of neutropenia with more than grade 1 of diarrhea, (4) grade 4 of anemia, (5) grade 4 or grade 3 of thrombocytopenia with transfusion needed, 6) grade 3 or worse of non-hematological adverse events persisting despite appropriate treatment (expected with electrolyte abnormalities).

The efficacy of FOLFIRINOX, tumor response, progression-free survival (PFS) and overall survival (OS) were evaluated only in patients with metastatic PC who were administered FOLFIRINOX as first-line therapy. Tumor response was assessed according to the response evaluation criteria in solid tumors (RECIST) version 1.1 [14].

### Statistical analysis

Mann–Whitney *U* test and Fisher’s exact test were used for comparing independent samples of quantitative and binary data, respectively. PFS was defined as the period from the start of first-line treatment to documentation of tumor progression or death. OS was defined as the period from the start of first-line treatment to death. Time-to-event data were analyzed using standard methods, including Kaplan–Meier product-limit estimates. Statistical analyses were performed using the JMP statistical software, Version 12.

## Results

### Patient characteristics and UGT1A1 polymorphism

A total of 31 patients from 16 institutions were enrolled. The patient characteristics are shown in Table 1. The median age at the initiation of FOLFIRINOX was 57 years (42–73). The ECOG PS was 0 or 1 in all patients. Twenty patients (65%) had metastatic disease, and 14 patients (45%) were diagnosed as having tumors in the pancreatic head. The genotypes of UGT1A1*28 and *6 were *6/*6 in 13 patients (42%), *28/*6 in 13 patients (42%), and *28 /*28 in 5 patients (16%). Patients who were administered FOLFIRINOX as first- and second-line therapies were in 26 (84%) and 3 (10%), respectively.

**Table 1:** Patient characteristics (*n* = 31)

		No. of patients (%)
Age (median, [range])		57 [42–73]
Sex	Male	20 (65)
ECOG PS	0	20 (65)
	1	11(35)
Extent of disease	Recurrence	5 (16)
	Locally advanced	6 (19)
	Metastatic	20 (65)
Location of the primary tumor	Head	14 (45)
Biliary drainage		10 (32)
CA19-9 (U/ml)	Median [range]	1,591 [1.2–27,400,000]
UGT1A1 genotype	*6/*6	13 (42)
	*28/*6	13 (42)
	*28/*28	5 (16)
No. of treatment cycles	Median [range]	8 [1–32]
Treatment line	1	26 (84)
	2	3 (10)
	≥ 3	2 (6)
Drug doses in the first cycle of FOLFIRINOX (mg/m^2^)		
Irinotecan	180	1 (3)
	150	5 (16)
	120	5 (16)
	90–100	11 (35)
	70–80	5 (16)
	≤ 60	4 (13)
Oxaliplatin	80–85	30 (97)
	60–65	1 (3)
5-FU continuous infusion	2200–2400	30 (97)
	1600–1800	1 (3)
G-CSF use	Present	12 (38)

The initial doses of each drug in the FOLFIRINOX regimen are described in Table 1. The initial dose of irinotecan varied widely from 30 to 180 mg/m^2^ (mean 90 mg/m^2^). The proportions of patients who received standard-dose oxaliplatin (85 mg/m^2^), 5-FU infusion (400 mg/m^2^), and 5-FU continuous infusion (2400 mg/m^2^) in the first cycle of FOLFIRINOX were 97, 19 and 97%, respectively. The median number of treatment cycles was 8 (1–32). Dose reduction of irinotecan to 70–100 mg/m^2^ was needed in five out of six patients (83%) who received the drug at the initial dose of 150 or 180 mg/m^2^ in the first cycle. The major reasons for discontinuation of the treatment were disease progression (88%) and adverse events (12%). The mean dose of irinotecan, oxaliplatin and 5-FU continuous infusion in all cycles of FOLFIRINOX were 91, 73 and 2242 mg/m^2^, respectively. G-SCF was administered to 12 patients (38%) during the entire course of FOLFIRINOX therapy, but none of the patients, except one, received the agent prophylactically during the first cycle of FOLFIRINOX therapy.

### Adverse events

Severe adverse events were observed in 8 out of 31 patients and there was no significant difference in the incidence depends on the initial dose of irinotecan. The main grade 3 or 4 adverse events were neutropenia (65%), FN (13%), and diarrhea (6%) (Table 2). There was no relationship between the incident of non-hematological grade 3/4 adverse events and dose of the initial dose of irinotecan in the first cycle of therapy (Table 3). We also assessed the frequency of Grade 4 neutropenia in 30 patients, after excluding 1 patient who received prophylactic G-CSF during the first cycle of FOLFIRINOX therapy. Grade 4 neutropenia was observed more frequently observed in patients who had received irinotecan at an initial dose of  ≥ 150 mg/m^2^ (67%; 4/6) than in those who had received the drug at an initial dose of  ≤ 120 mg/m^2^ (20%; 5/24) (Table 3).

**Table 2:** Adverse events

Adverse events, *n* (%), (*n* = 31)	Toxicity grade	
	Grade ≥ 3	All grade
Neutropenia	20 (65)	23 (74)
Thrombocytopenia	3 (10)	16 (52)
Febrile neutropenia	4 (13)	4 (13)
Diarrhea	2 (6)	17 (55)
Anorexia	5 (16)	21 (68)
Fatigue	2 (6)	19 (61)
Nausea	1 (3)	15 (48)
Vomiting	1 (3)	6 (19)
Peripheral neuropathy	2 (6)	16 (52)

**Table 3:** Grade 3 and 4 of adverse events stratified by the initial dose of irinotecan in the first cycle of treatment

Variable, *n* (%), (*n* = 31)	Initial dose of irinotecan in the first cycle of treatment				
	≥ 150 mg/m^2^	120 mg/m^2^	90–100 mg/m^2^	70–80 mg/m^2^	≤ 60 mg/m^2^
Neutropenia	4 (67)	1 (20)	3 (27)	1 (20)	0
Thrombocytopenia	0	0	0	0	0
Febrile neutropenia	1 (17)	0	2 (18)	1 (20)	0
Diarrhea	0	0	2 (18)	0	0
Anorexia	2 (33)	0	2 (18)	0	1 (25)
Fatigue	1 (17)	1 (20)	0	0	0
Nausea	0	0	1 (9)	0	0
Vomiting	0	0	1 (9)	0	0
Peripheral neuropathy	0	0	1 (9)	0	1 (25)

### Efficacy

**Fig. 1:**
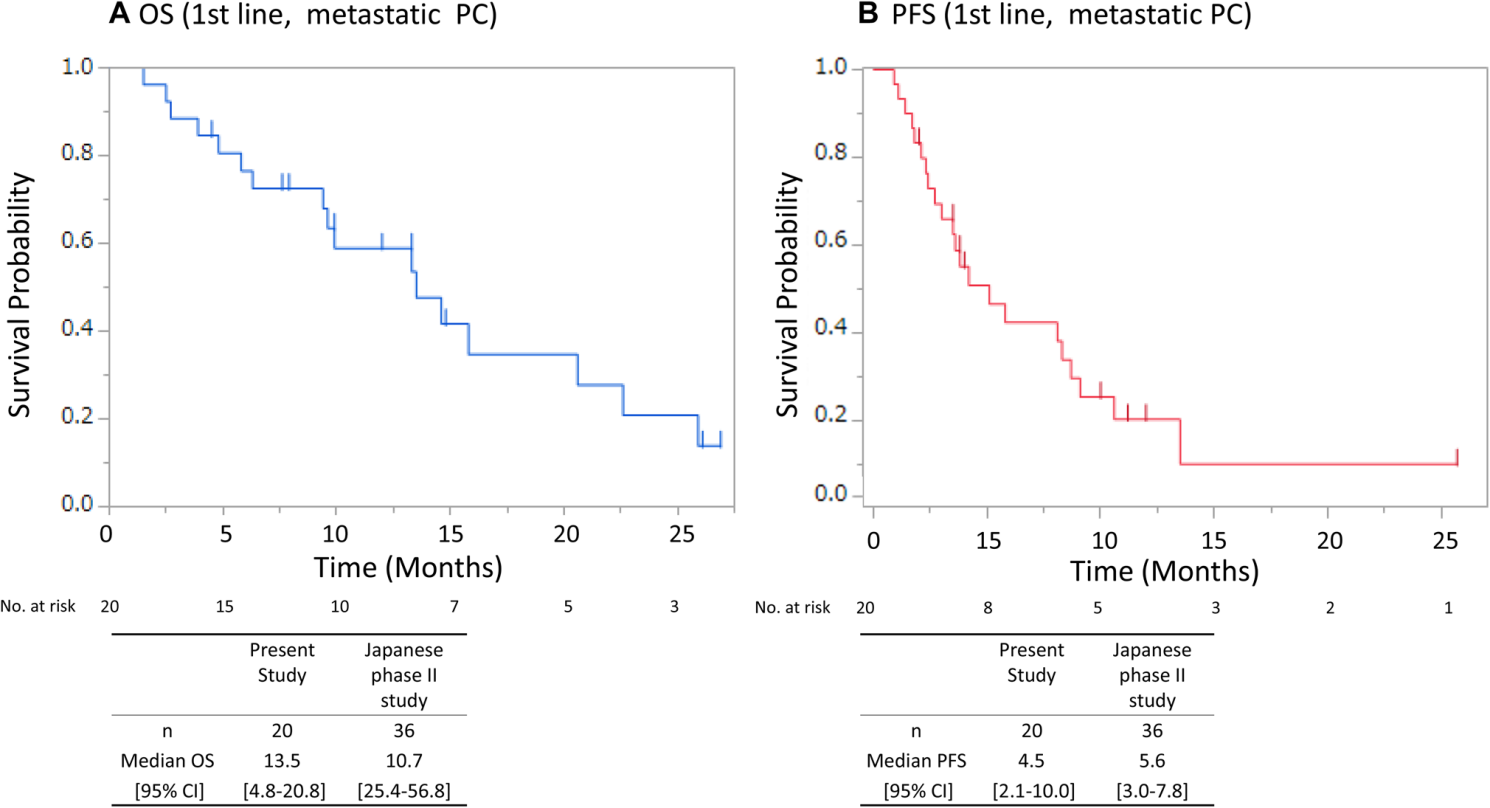
Overall survival (**a**) and progression-free survival (**b**) in patients with metastatic PC administered FOLFIRINOX as first-line treatment. *OS* Overall survival, *PFS* progression-free survival

**Fig. 2:**
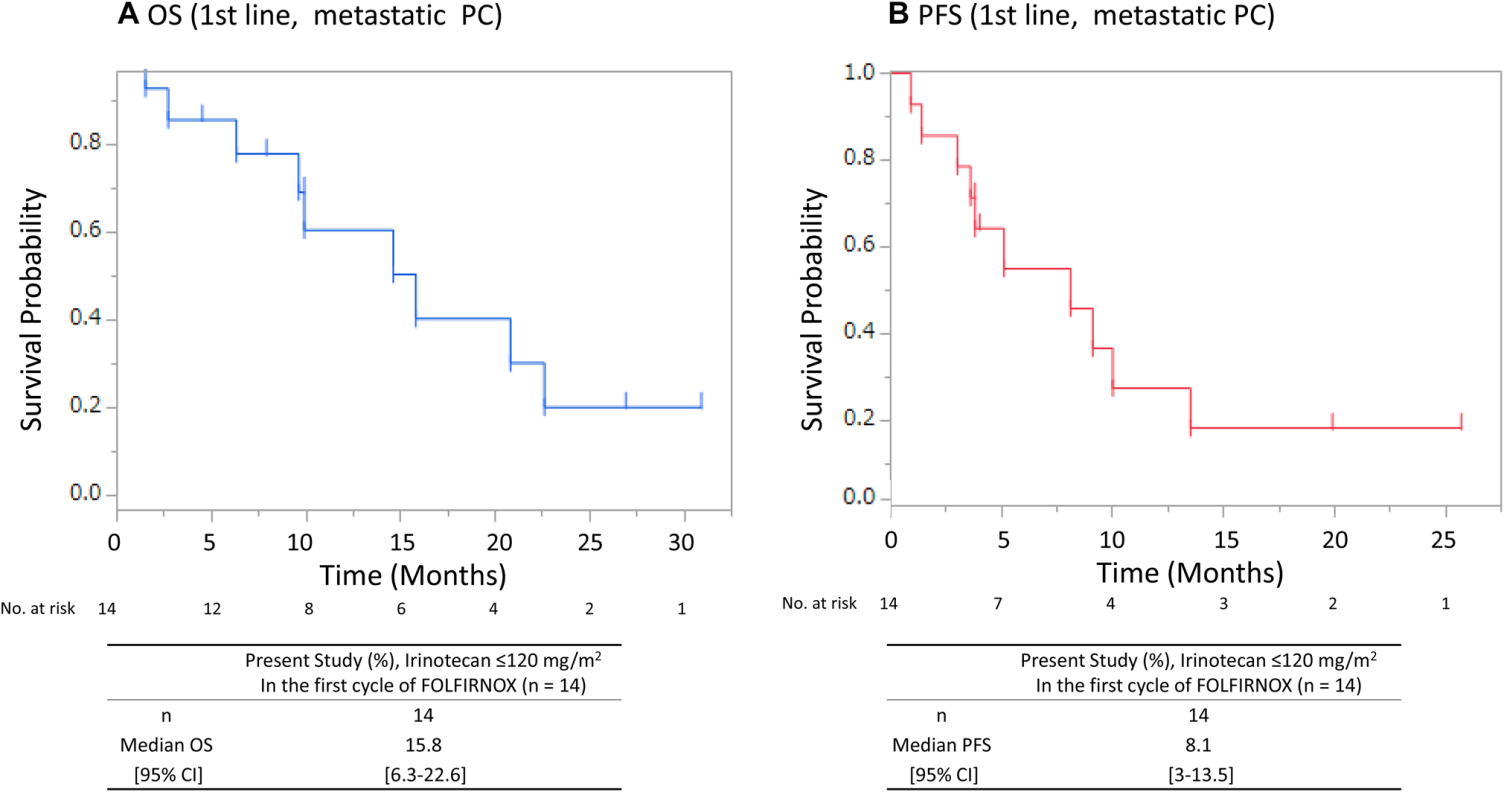
Overall survival (**a**) and progression-free survival (**b**) in patients with metastatic PC given irinotecan at the starting dose of ≤120 mg/m2 during the first cycle of FOLFIRNOX as first-line treatment. *OS* Overall survival, *PFS* progression-free survival

The median follow-up time was 9.9 months. With regard to the tumor responses as assessed by RECIST 1.1, the responses were classified as partial response, stable disease, progressive disease, and not evaluable (NE) in 20.0, 50.0, 25.0, and 5% of the enrolled patients, respectively. The RR was 20% (95% CI, 6–43), and the disease control rate (DCR) was 70% (95% CI, 36–80) in patients with metastatic PC who received FOLFIRINOX as first-line therapy. The median OS in patients with metastatic PC who received FOLFIRINOX as first-line treatment was 13.5 months (95% CI 4.8–20.8), and the median PFS was 4.5 months (95% CI 2.1–10.0) (Fig. 1). We also assessed the efficacy in metastatic PC patients who received FOLFIRINOX of first-line treatment, with irinotecan at the initial dose of  ≤ 120 mg/m^2^; the median OS was 15.8 months (95% CI 6.3–22.6), the median PFS was 8.1 months (95% CI 3.0–13.5) (Fig. 2), the RR was 21.4% (95% CI 10.2–32.6) and the DCR was 85.7% (95% CI 13.1–40.9).

## Discussion

Irinotecan-based regimens are one of the key therapies in advanced PC patients, and global phase III [8] and domestic phase II [9] studies have already shown the safety and efficacy of FOLFIRINOX. However, there have been no studies investigating the influence of UGT1A1-DV, especially both *28 and *6 morphism, in Japanese patients with advanced PC.

In terms of adverse events, the incidences of grade 3 or 4 of non-hematological toxicities, such as diarrhea, were consistent with the result of previous Japanese phase II study of mFOLFIRINOX [10] conducted in non-UGT1A1-DV patients. However, grade 4 of neutropenia was more frequently observed in patients who received a high initial dose of irinotecan (≥ 150 mg/m^2^) in the first cycle as compared to those who received lower initial dose (≤ 120 mg/m^2^) (Table 4). Grade 4 neutropenia is a critical adverse event and the best effort should be made to avoid its occurrence; patients with Grade 4 neutropenia are frequently hospitalized with fever and are at a risk of death from neutropenic sepsis. In addition, patients who received irinotecan at a higher initial dose more often needed dose reduction, with the dose finally reduced to 70–100 mg/m^2^. Irinotecan administered at an initial dose of  ≥ 150 mg/m^2^ seemed to be highly toxic. From the results of the present study, we proposed that the initial dose of irinotecan in FOLFIRINOX regimen should be  ≤ 120 mg/m^2^. Satoh et al. [15] reported that the maximum limiting dose was 150 mg/m^2^ in biweekly irinotecan monotherapy for metastatic gastrointestinal cancer patients with UGT1A1-DV. Kim et al. [16] also planned triweekly irinotecan plus capecitabine for metastatic colorectal cancer patients with UGT1A1-DV and concluded that the maximum limiting dose was 200 mg/m^2^. These previous reports focused on patients with UGT1A1-DV, however, but did not assess patients with PC treated with FOLFIRINOX. In Japan, wild and heterozygous type of UGT1A1*6 and *28 polymorphism in patients with unresectable PC were assessed by Shirasu et al. [17]. There was no difference in the frequency of adverse events depending on UGT1A1 status in patients who received mFOLFIRINOX.

**Table 4:** Hematological adverse events (grade 4)

Variable, *n* (%), (*n* = 30)	Initial dose of irinotecan in the first cycle of treatment				
	≥ 150 mg/m^2^	120 mg/m^2^	90–100 mg/m^2^	70–80 mg/m^2^	≤ 60 mg/m^2^
Neutropenia	4 (67)	1 (25)	3 (27)	1 (20)	0
Thrombocytopenia	0	0	0	0	0

Efficacy in patients with metastatic PC administered FOLFIRINOX as a first-line treatment was associated with the RR of 20.0%, the median PFS of 4.5 months, and the median OS of 13.5 months. These results were not different from those reported from a Japanese phase II study of FOLFIRINOX, and were also consistent with those reported in patients given irinotecan at an initial dose of  ≤ 120 mg/m^2^ in the first cycle of FOLFIRINOX (RR: 21.4%, median PFS: 8.1 months and median OS: 15.8 months).

On the basis of these results, we suggested that an initial dose of irinotecan  ≤ 120 mg/m^2^ is possibly the optimal dose for the first cycle of FOLFIRINOX for Japanese advanced PC patients with UGT1A1-DV. This study had some limitations. First, irinotecan was administered at various doses and each group of patients divided by the dose of irinotecan had only a few cases. Therefore, we could not adequately assess the differences in the clinical outcomes among these groups in this study. Second, no patients except one received the G-CSF prophylactically during the first cycle of FOLFIRINOX therapy. Therefore, the potential impact of prophylactic G-CSF on the incidence of Grade 4 neutropenia is unknown in patients with UGT1A1-DV. Third, we calculate the mean dose, but not the relative dose intensity (RDI). Therefore, a prospective study would be needed in the future to determine RDI for validating the adverse events and efficacy of FOLFIRINOX for patients with UGT1A1-DV. Sharma et al. [18] also conducted a phase I study of FOLFIRINOX for pancreatic, biliary and gastric cancer patients with homozygous only for UGT1A1*28 and showed a high frequency of adverse events, such as neutropenia; they concluded that irinotecan 90 mg/m^2^ cannot be considered as the recommended dose. Further study is warranted to evaluate the safety, feasibility and efficacy for FOLFIRINOX in advanced PC patients with UGT1A1* both 6 and *28-DV.

In conclusion, the incidence of neutropenia in this study indicated that advanced PC patients with UGT1A1-DV tolerate irinotecan administered at the initial dose of  ≤ 120 mg/m^2^. The efficacy in patients given irinotecan at the initial dose of  ≤ 120 mg/m^2^ in this study was consistent with previous reports.
